# High‐Precision Photoacoustic Neural Modulation Uses a Non‐Thermal Mechanism

**DOI:** 10.1002/advs.202403205

**Published:** 2024-06-26

**Authors:** Guo Chen, Feiyuan Yu, Linli Shi, Carolyn Marar, Zhiyi Du, Danchen Jia, Ji‐Xin Cheng, Chen Yang

**Affiliations:** ^1^ Department of Electrical and Computer Engineering Boston University Boston MA 02215 USA; ^2^ Department of Chemistry Boston University Boston MA 02215 USA; ^3^ Department of Biomedical Engineering Boston University Boston MA 02215 USA

**Keywords:** ion channels, neuromodulation, patch clamp, photoacoustic

## Abstract

Neuromodulation is a powerful tool for fundamental studies in neuroscience and potential treatments of neurological disorders. Both photoacoustic (PA) and photothermal (PT) effects are harnessed for non‐genetic high‐precision neural stimulation. Using a fiber‐based device excitable by a nanosecond pulsed laser and a continuous wave laser for PA and PT stimulation, respectively, PA and PT neuromodulation is systematically investigated at the single neuron level. These results show that to achieve the same level of neuron activation recorded by Ca^2+^ imaging, the laser energy needed for PA stimulation is 1/40 of that needed for PT stimulation. The threshold energy for PA stimulation is found to be further reduced in neurons overexpressing mechano‐sensitive channels, indicating direct involvement of mechano‐sensitive channels in PA stimulation. Electrophysiology study of single neurons upon PA and PT stimulation is performed by patch clamp recordings. Electrophysiological features induced by PA are distinct from those by PT, confirming that PA and PT stimulation operate through different mechanisms. These insights offer a foundation for the rational design of more efficient and safer non‐genetic neural modulation approaches.

## Graphical abstract

Understanding the mechanisms of different non‐electrical neurostimulation methods offers new insights for neuroscience and enables more efficient and safer clinical treatments. Here, using a fiber emitter (FE), this work shows that photoacoustic stimulation is 40 times more efficient than photothermal stimulation. The electrical response from photoacoustic stimulation is distinct from photothermal stimulation. Mechano‐sensitive channels are involved in photoacoustic stimulation.

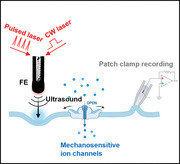

## 1 Introduction

Neuromodulation with high spatial precision is a valuable tool for understanding the flow of information in the neural systems and treatment of neurological disorders. Non‐electromagnetic neuromodulation developed in the past two decades has added many options to this toolkit. Insights gained on the cellular mechanism of these non‐electrical methods deepen our understanding of neuroscience and facilitate the rational design of methods for clinical applications. For example, optogenetics allows control over the activity of selected cells using a combination of genetic engineering and light. Some promising clinical potential for optogenetics is emerging, for example, in treating retinal degenerative disease.^[^ ^1^ ^]^ However, broader clinical use is limited, as it requires genetic manipulation.^[^ ^2^ ^]^ Focus ultrasound has been demonstrated as a non‐genetic non‐invasive brain modulation method and has been tested in multiple clinical trials. Emerging evidence suggests that mechanosensitive ion channels on the neuron membrane are involved in ultrasound stimulation.^[^ ^3^ ^]^ Yet its spatial resolution is typically a few millimeters.

Non‐genetic optical stimulation offers a promising solution to address the abovementioned limitations. Infrared neurostimulation uses the photothermal (PT) effect to trigger neuronal activities.^[^ ^4^ ^]^ Strong light‐absorbing nanomaterials, including gold nanoparticles,^[^ ^5^ ^]^ Si nanowires,^[^ ^6^ ^]^ polymers^[^ ^7^ ^]^ and carbons,^[^ ^8^ ^]^ were used to enhance the thermal effect. Two main PT stimulation mechanisms have been suggested. The first is a direct thermal mechanism, where a temperature increase of a few degrees, induced by light, could activate thermosensitive ion channels.^[^ ^9^ ^]^ Yet, the substantial increase in temperature raises significant safety concerns, particularly in clinical applications. The second is an opto‐capacitive mechanism, which requires a rapid and transient temperature increase at a rate of kilokelvins per second upon light irradiation. This modulates the capacitance of the cell membrane,^[^ ^10^, ^11^ ^]^ and drives a sufficient transmembrane capacitive current.^[^ ^12^ ^]^ Yet, such steep slope of temperature increase precludes its application in vivo due to the challenge to focus a pulsed laser through scattering tissues.

Recently, the photoacoustic (PA) effect has been harnessed as a new non‐genetic approach for high‐precision neuromodulation.^[^ ^13^ ^]^ In 2020, Jiang et al. reported the development of a fiber optoacoustic emitter,^[^ ^14^ ^]^ capable of sub‐millimeter neurostimulation both in vitro and in vivo. Sub‐millimeter precise stimulation of rodent brains in the somatosensory and motor cortex has been demonstrated. In addition to the fiber device, other modalities were also developed for different applications, including biocompatible optoacoustic films for neural stimulation and regeneration,^[^ ^15^ ^]^ photoacoustic nanotransducers toward minimal invasive brain stimulation,^[^ ^16^ ^]^ and optically generated focused ultrasound (OFUS) for non‐invasive transcranial brain stimulation.^[^ ^17^ ^]^ Photoacoustic neural stimulation is an emerging technology for high‐precision, nongenetic, safe neural stimulation.^[^ ^18^ ^]^

Despite these technical advances, the mechanism of PA stimulation remains intriguing. Physically, in a PA process, absorption of pulsed light results in local and transient heating, inducing thermal expansion of the absorber or medium and leading to a propagating mechanical wave at the ultrasound frequencies.^[^ ^19^ ^]^ When the PA emitters are placed near the targeted neurons, the neurons are expected to sense both the pressure wave from the PA effect and the transient temperature rise due to heat generated by the PA emitters. Notably, the energy conversion efficiency of a typical photoacoustic material is less than 3%.^[^ ^19^, ^20^ ^]^ Therefore, it is crucial to dissect which process plays a major role in triggering the neural activity and to find out the mechanism that boosts the efficiency of PA neuromodulation.

In this study, we first demonstrate a universal fiber‐based device that can be used for both PA and PT stimulation at the single‐neuron level. The fiber device acts as a PA or PT emitter driven by corresponding laser conditions. On the recording side, while conducting simultaneous whole‐cell patch clamp recordings is highly challenging in neuronal stimulation by transducer ultrasound, it is feasible upon fiber‐based PA and PT stimulation. With this tool, we systematically investigated and compared PA and PT neuromodulation under different laser conditions. The results reveal that the laser energy required for achieving the same level of activation in PA stimulation is ∼1/40 of that needed for PT stimulation. Studies on temperature changes in neurons show that PA stimulation is associated with negligible temperature rise, confirming its non‐thermal mechanism. Ruling out the thermal effect, we further studied the molecular mechanisms for PA neuromodulation. Through overexpressing and pharmacologically blocking of ion channels, mechanosensitive ion channels including TPRC1, TRPP2, and TRPM4 were identified to play significant roles in boosting the stimulation effect. Electrophysiology study of single neurons upon PA and PT stimulation was performed by patch clamp recordings. Electrophysiological features stimulated by PA are distinct from those induced by PT, further confirming that PA and PT stimulation‐ operate through distinct mechanisms.

## 2 Results

### 2.1 A Fiber Device for PA and PT Stimulation

**Figure 1:**
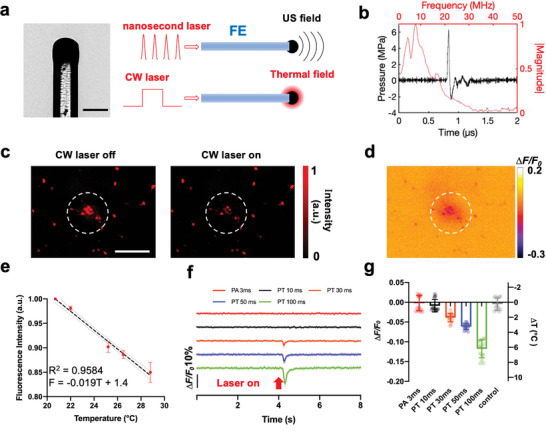
FE used for both PT and PA stimulation and its thermal effect measured in the neurons labeled with mCherry. a) Picture and schematic of FE generating photoacoustic and photothermal signals using different lasers. Scale bar: 200 µm. b) Representative photoacoustic signal generated by FE using the nanosecond pulsed laser. Black: waveform in the temporal region. Red: frequency spectrum. Laser energy: 45 µJ per pulse. c) Representative mCherry fluorescence imaging of neurons under FE before and after CW laser is on. Laser condition: 1064 nm CW laser, 120 mW, 100 ms duration. Scale bar: 200 µm. d) Contrast image showing the decrease of fluorescence intensity of the same view field in (c). Dashed circles: area of the FE. e) Calibration curve of the normalized mCherry fluorescence intensity change due to temperature increase. Standard deviation (SD) was taken from five neurons. f) Average mCherry fluorescence traces taken from 15 neurons under the FE applied with different laser conditions. Laser condition for PA: the nanosecond laser, 120 mW, repetition rate 4.23 kHz. Laser condition for PT: the CW laser, 120 mW. g) Average minimum *∆F/F_0_* of the 15 mCherry labeled neurons in f and estimated temperature change under different conditions. Control: no laser.

To directly compare the PA and PT stimulation at the single cell level, we designed a fiber emitter (FE) to produce the PA or PT effect selectively by coupling it to different excitation lasers. Specifically, the tip of a commercial optical fiber with a diameter of 200 µm was coated with a layer of candle soot, as an absorber, and a second layer of polydimethylsiloxane (PDMS), as described previously.^[^ ^21^ ^]^ According to the PA theory,^[^ ^22^ ^]^ under irradiation by a nanosecond pulsed laser, both thermal and stress confinement conditions are met for efficient PA conversion. The same FE was connected to a nanosecond (ns) pulsed laser (RPMC One 100uJ–1030 nm) for PA stimulation and to a continuous wave (CW) laser (Cobolt Rumba, 1064 nm) for PT stimulation (**Figure** **1**a).

Under the pulsed laser condition, the FE efficiently emits a PA signal measured by a needle hydrophone with a diameter of 40 µm (Precision Acoustic, UK) (Figure 1b). The hydrophone was aligned with the FE and placed 50 µm away from the tip of the FE (Figure S1, Supporting Information). Under the laser condition of 45 µJ per pulse, the FE produced an ultrasound waveform with a peak‐to‐peak pressure of over 8 MPa (Figure 1b), which is consistent with previously published results and is sufficient for efficient PA neural stimulation.^[^ ^21^ ^]^ When coupled with the CW laser, the FE functions as a pure PT source and generates a very localized thermal field visualized by a thermal camera (Figure 1a; Movie S1, Supporting Information). Together, owing to the strong absorption of the candle soot and the high thermal expansion coefficient of PDMS,^[^ ^23^ ^]^ the FE serves as a point source of ultrasound and a source of heat under specific laser conditions.

### 2.2 Neuron Temperature under PT and PA Conditions

To directly measure the thermal effect of the FE on neurons under PA and PT conditions, we monitored the temperature change on neurons using the fluorescence of mCherry as a sensitive temperature indicator.^[^ ^3^ ^]^ mCherry was delivered to neurons via AAV9 viral vector transfection (Addgene, pAAV‐hSyn‐mCherry). Through fluorescence imaging, we confirmed the reduction in fluorescence of mCherry when temperature increases. As depicted in Figure 1c,d, when applying the CW laser with a duration of 100 ms and an average power of 120 mW to the FE, the fluorescence intensity of mCherry exhibited a decrease of over 10%. The FE was placed within 50 µm away from the neurons.

We then performed a calibration experiment by imaging mCherry‐labeled neurons at different temperatures. The temperature of the neurons was raised in the range from 20 to 30 °C in a controlled manner using a dish heater and monitored by a thermal coupler in the medium. For each temperature, the medium was heated until the temperature stabilized at the targeted temperature for over 20 s. Figure 1e plots the normalized fluorescence intensity of mCherry as a function of temperature measured by the thermal coupler. For each data point, five cells in the field of view were selected and the fluorescence intensity of the cell was determined by averaging all pixels from the cell. The fluorescence excitation light was only turned on after the temperature stabilized and was turned off immediately after capturing the fluorescence image to minimize any possible photobleaching that might affect the fluorescence intensity. A linear fit was applied to calibrate the intensity of mCherry fluorescence to the temperature. The results indicate that the fluorescence intensity decreased by 1.9% for every 1 °C increase in temperature (Figure 1e), consistent with previously published results.^[^ ^3^ ^]^

Based on the calibration, we evaluated the temperature change on the mCherry‐labeled neurons (*n* = 15) under a wide range of laser conditions used for PA and PT stimulation. The FE was placed at a controlled distance of less than 50 µm away from a neuron. Temperature changes were calculated based on the fluorescence change of mCherry and the calibration curve. Under the FE with a 10 ms duration of the CW laser at a power of 120 mW, no significant fluorescence change was observed, and thus the temperature increase is negligible (Figure 1f black line, −0.01 ± 0.02 °C). For PT conditions with longer laser durations, a significant drop in fluorescence intensity was observed upon laser activation. Specifically, with the CW laser durations of 30, 50, and 100 ms, the corresponding temperature increase was calculated to be 2.0 ± 0.6, 3.2 ± 0.4, and 6.2 ± 1.2 °C (Figure 1g orange, blue, and green), respectively.

We also performed mCherry temperature measurements under PA conditions. Based on previously reported studies,^[^ ^21^ ^]^ the PA signal generated by a burst of nanosecond pulsed laser of 3 ms is sufficient to activate neurons. Here, maintaining the average laser power at 120 mW, the same as the PT laser condition, a pulse train (1030 nm, 3 ns, RPMC, Fallon, MO, USA) of 3 ms duration was applied to the FE and the fluorescence intensity of mCherry was monitored (Figure 1f, red curve). A temperature increase detected was negligible, comparable to the control conducted with no laser (Figure 1g). Notably, the fluorescence imaging of mCherry was conducted with a 20 Hz imaging rate. While this imaging speed was insufficient to precisely track the transient heating associated with the ns laser pulses or to capture the fast rising of the temperature during the 3 ms laser duration, the cooling process was sufficiently slow to be recorded through the fluorescence imaging. Notably, the slow decay is indeed presented in the PT groups shown in Figure 1f. The distinct decay features measured suggested that PA has minimal temperature change, compared to the PT conditions. Collectively, by the temperature measurements under different laser conditions, the PA groups showed a clear difference from the PT groups. Specifically, the temperature rise on neurons is negligible under the pulsed laser condition.

### 2.3 PA Stimulation Uses Much Lower Laser Energy Than PT Stimulation

To determine the laser energy input needed for PA and PT stimulation, we deployed calcium imaging to monitor the neural activities under different laser conditions. Although widely used to monitor neuronal activity, GCaMP was reported to show a high sensitivity to temperature, which consistently resulted in significant thermal artifacts during PT experiments.^[^ ^24^ ^]^ Thus, we opted to employ OGD‐488 (Oregon Green 488) as a calcium indicator. OGD‐488 exhibits lower sensitivity to thermal influences and allows more accurate observation of neuronal activity.

**Figure 2:**
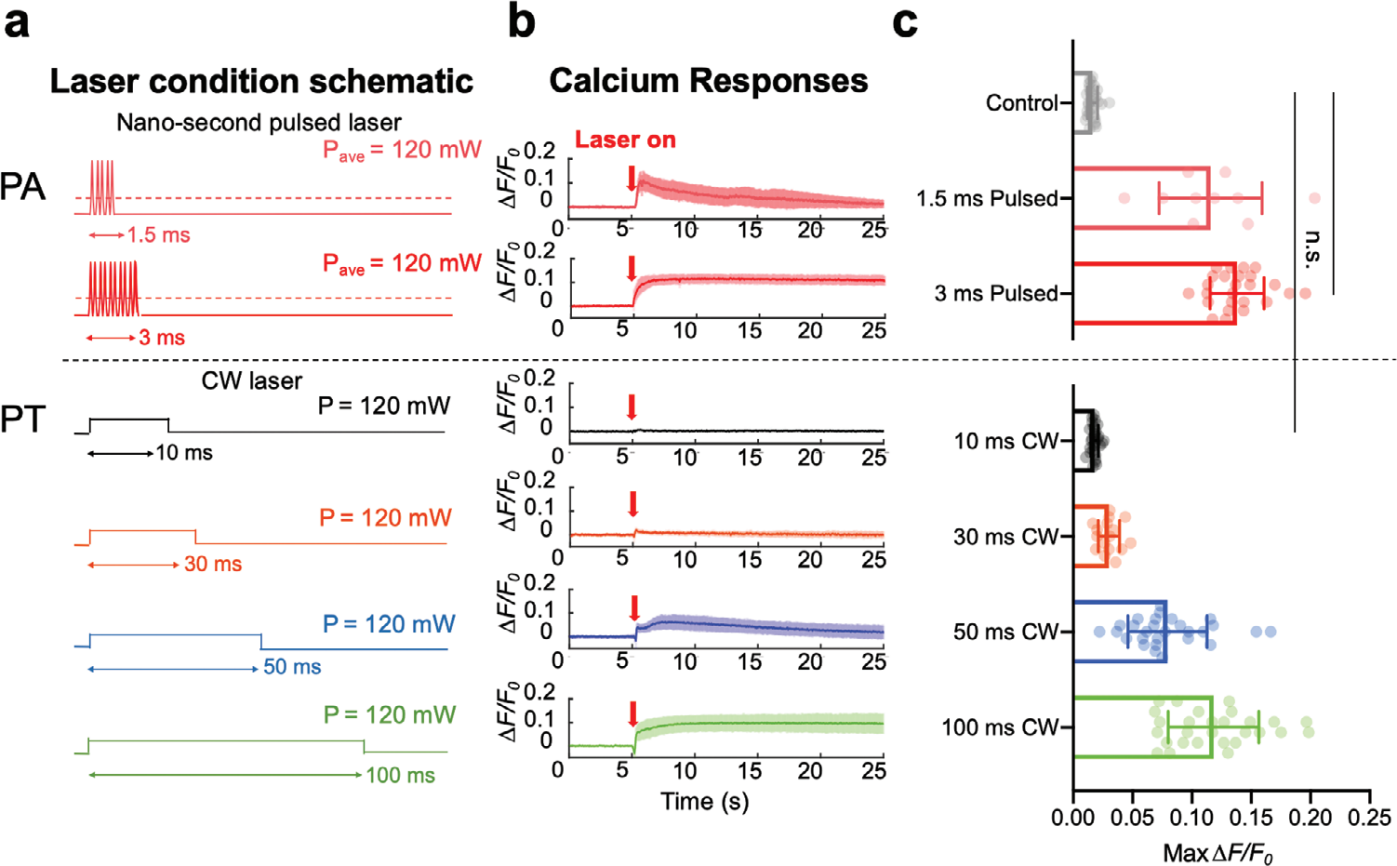
Comparison of neuron response upon PA and PT stimulation recorded by Ca^2+^ imaging. a) Schematic of different laser conditions used in PA/PT stimulation. Dark pink: pulsed laser with 5 pulses in 1.5 ms. Red: pulsed laser with 11 pulses in 3 ms. Black: CW laser with 10 ms duration. Orange: CW laser with 30 ms duration. Blue: CW laser with 50 ms duration. Green: CW laser with 100 ms duration. Average power of all laser conditions: 120 mW. b) Ca^2+^ traces of neurons under different laser conditions shown in a. Laser on at t = 5 s (Red arrows). Solid lines: averaged traces. Shaded areas: SD. c) Statistical analysis of maximum Δ*F*/*F*0 of neurons under PA and PT stimulation. *n* = 28, 10, 25, 28, 16, 28 for 1.5 ms pulsed, 3 ms pulsed, 10 ms CW, 30 ms CW, 50 ms CW, 100 ms CW, respectively. Control: no laser. *n* = 28 for control group, *t‐test*, ^****^ *p* < 0.0001. n.s. no significance.

First, we applied the 1030 nm pulsed laser with an average power of 120 mW and a repetition rate of 4.23 kHz to an FE placed within 50 µm of the neuron. Our earlier study confirmed that the effective stimulation area is within 100 µm of the FE used; therefore, the FE being positioned around 50 µm away from the targeted neurons ensures efficient stimulation.^[^ ^21^ ^]^ We estimated the pressure generated by the FE is 5 MPa, based on the laser pulse energy. When the laser duration was 1.5 ms, corresponding to five 3 ns laser pulses, a transient activation was observed (Max Δ*F*/ *F*_0_ =  11.7 ± 4.3%) (**Figure** **2**a,b, dark pink traces). When the laser duration was increased to 3 ms, i.e., eleven laser pulses, the responses from the neurons were stronger and the averaged fluorescence change reached 13.9 ± 2.3% (Figure 2a,b, red traces). The number of laser pulses was confirmed by a photodiode (Figure S5, Supporting Information). Fluorescence images can be found in Figure S2a,b (Supporting Information).

We then applied the 1064 nm CW laser to the same FE. The CW laser was operated at the same average power of 120 mW (Figure 2a), acting as a pure PT source. The absorption spectrum of the candle soot under both the 1030 and 1064 nm was shown to be the same (Figure S3, Supporting Information). Therefore, the total energy absorbed by FE under these two laser conditions is expected to be the same.

Unlike the pulsed laser condition, employing a CW laser of 10 ms duration with the same average power failed to elicit neuronal activities within the area of interest, indicated by the minimal fluorescence change (1.8 ± 0.3%). (Figure 2b, black trace). This result shows that even though we delivered nearly three times the laser energy to the FE, the PT stimulation threshold was not reached. When the CW laser duration increased to 30 ms, a fluorescence response of 3.0 ± 0.9% was observed (Figure 2b, orange trace). Notably, when increasing the CW laser duration to 50 ms, the fluorescence change reached 7.9 ± 3.3% and clear activation of the neurons was observed (Figure 2b, blue trace). Finally, with the CW laser duration reaching 100 ms, the neurons were effectively stimulated (11.8 ± 3.8%), displaying a fluorescence trace akin to that observed under the 3 ms photoacoustic condition (Figure 2b, green trace). Notably, a transient signal decrease in the Ca^2+^ trace was observed in the 50 and 100 ms PT stimulation groups. It is a thermal artifact associated with the temperature increase when the laser is turned on.^[^ ^25^ ^]^ In comparison, in the 3 ms PA stimulation group, the thermal‐induced fluorescence decrease was not observed, again confirming that temperature increase is negligible. We further conducted a statistical *t‐test* analysis to support our findings (Figure 2c). Specifically, no significant difference was found between the control group without light and the 10 ms PT group (n.s., *p* = 0.172). However, a significant difference was observed between the 3 ms PA group and the 10 ms PT group (^****^ *p* < 0.0001). Based on this comparative analysis, it becomes evident that the heat or temperature increase generated under the 3 ms pulsed laser condition is insufficient to stimulate neurons. Considering the first 0.6 ms of idle time when the pulsed laser was turned on (Figure S5, Supporting Information), effectively, only 2.4 ms was needed for PA stimulation, compared to the 100 ms laser duration needed for PT stimulation. These results suggest that the energy dosage needed from PA stimulation is only ‐∼1/40 of that for PT stimulation when achieving a similar level of neuron activation.

### 2.4 Patch Clamp Recording Shows Distinct Neuronal Response to PA and PT Stimulation

**Figure 3:**
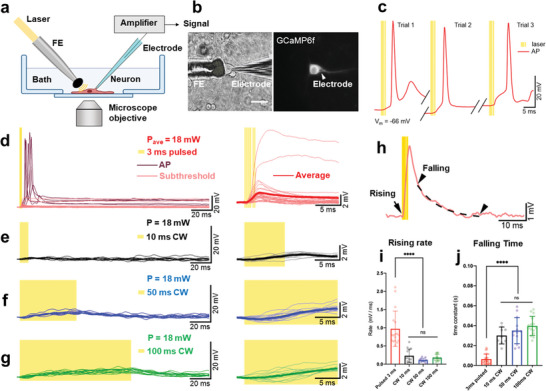
PA‐triggered subthreshold depolarizations and action potentials (APs) are distinct from PT effects. a) Schematic of tapered FE stimulation and electrophysiological recording. b) Transmission and fluorescence images of simultaneous whole cell patch clamp and tapered FE stimulation on the same neuron. Left: transmission image. Right: GCaMP6f fluorescence image. Scale bar: 50 µm. c) Representative APs triggered by PA stimulation in three consecutive trials on one neuron. Each yellow line denotes a 3 ns laser pulse. d) Overlaying representative single trial recordings of membrane responses, including APs and subthreshold membrane depolarizations, induced by 3 ms pulsed laser (*n* = 41, from 6 cells). Dark red traces: APs. Pink traces: subthreshold depolarizations (*n* = 27, 4 cells). Yellow shaded area denotes pulsed laser on. Right panel: Same subthreshold depolarization traces at the early stage of depolarizations. Bold red line: average of all the subthreshold depolarizations. e–g) Overlaying representative single traces of membrane responses triggered by CW laser of 10, 50, and 100 ms duration (*n* = 17, from 3 cells). Yellow shaded area denotes laser on. Right panel: the same traces show the beginning of the depolarizations. Bold lines: average of the traces in each condition. All traces in (d–g) were normalized to the same baseline at 2 ms before laser delivery. h) An example trace from panel d indicating the selected time points for measurements in (i) and (j). The black arrow denoted the selected time point when the rising rate in (i) was measured. Two arrow heads point out the start and end of the selected period for calculating the time constant in the decay phase in (j). Dashed line: single exponential fit of the selected curve between the arrow heads. i) Statistical summary of the rising rate of the subthreshold depolarizations at the very beginning in respond to FE stimulation. *ANOVA* test. ^****^ *p* < 0.0001. j) Statistical comparison of the time constant of the decay phase in PA and PT‐triggered subthreshold depolarizations. *ANOVA* test. ^****^ *p* < 0.0001. APs in (d) were excluded from statistical analysis in (i) and (j) for comparison of only subthreshold events.

While it showed distinct energy dose requirements for PA and PT stimulation, Ca^2+^ imaging is an indirect measurement of neuronal activities. Instead, patch clamp recording can provide direct recording of sub‐ and supra‐threshold neuron activities. Previously, patch clamp recording of ultrasound stimulation has been limited as conventional transducer‐generated ultrasound easily disrupts the patch attachment. One unique capability of our device is its compatibility with whole‐cell patch‐clamp recording of single neurons.^[^ ^26^ ^]^ Here, we evaluated FE‐invoked electrical responses in cultured single cortical neurons with whole‐cell patch clamp (**Figure** **3**a). To be compatible with the patch‐clamp recording system, we used a tapered FE with a tip diameter smaller than 50 µm (Figure 3b).^[^ ^26^ ^]^ It can generate an ultrasound field confined to about 80 µm^[^ ^26^ ^]^ and enable single‐neuron stimulation in a low‐density neuron culture (<20 cells mm^−2^). Notably, the size of the fibers and laser power used here were chosen to be compatible with patch clamp measurement. They are different from what was used for the Ca^2+^ imaging studies, resulting in different response dynamics from that observed in the Ca^2+^ imaging. The nanosecond pulsed laser at 1030 nm with a 4.23 kHz repetition rate or the CW laser at 1064 nm was delivered to the tapered FE. The average power of each type of laser delivered at the tapered tip was measured to be 18 mW (*n* = 10).

As shown in Figure 3a (red line), the FE with a ns pulse train of 3 ms duration could directly trigger action potential (AP) firing in consecutive trials on single neurons (Figure 3c). It appears that each laser pulse could induce a small depolarization, gradually accumulating to reach the AP firing threshold with an average delay of 3.99 ± 3.29 ms after laser onset (Figure 3c, dark red curves in 3d). The difference in AP waveforms observed in Figure 3c is attributed to the small variations in the maximum power and delivery time of each laser pulse (Figure S5, Supporting Information). Sometimes, a failed second AP occurred (Figure 3c trial 1 and 3). Among the neurons tested (*n* = 19 from 5 animals), 58% responded to FE‐based PA stimulation. Among those who responded to FE stimulation, 36% responded with the occurrence of AP, presumably due to a lower laser energy dosage compared to the previous calcium imaging experiments, and resulted in a stimulation power around or below the threshold of AP firing.

Additionally, the characteristics of the PA‐triggered APs are found similar to those of APs that occurred spontaneously (Figure S4a–c, Supporting Information), including maximum amplitude, after‐hyperpolarization, AP half‐width, rising rate, and falling rate (Figure S4f,g, Supporting Information). We also compared them with electrically elicited APs (Figure S4d,e, Supporting Information). While resting membrane potential, maximum amplitude, and after‐hyperpolarization did not show statistically significant differences among the three groups, the electrically triggered APs exhibited a notably larger half‐width, slightly higher rising rate, and slower falling rate due to the differences in stimulation power and durations compared to PA elicited APs.

In most other responding cases, the same pulsed laser repeatedly triggered subthreshold depolarizations with an average amplitude of 1.84 ± 0.98 mV (Figure 3d, pink curves). The largest triggered amplitude was 13.57 mV while the smallest was 0.16 mV. The variations were likely caused by differential cell sensitivity to PA stimulation. The maximum amplitude occurred at 0.36 ± 0.66 ms after the laser offset on average. Most PA‐triggered subthreshold depolarizations initiated after 2–7 laser pulses, ∼0.2–1.1 ms, after the first laser pulse onset (Figure S5, Supporting Information). These data suggest that like spontaneous firing, ion channels are likely involved in PA stimulation.

In contrast to the PA condition, no APs were triggered by the FE driven by the CW laser of the same power of 18 mW, for durations up to 100 ms. The 10 ms CW laser‐induced membrane depolarizations with a maximum amplitude of 0.93 ± 0.40 mV at 2.90 ± 0.19 ms after laser offset (Figure 3e). Membranes of the same neurons were depolarized for 2.57 ± 0.24 mV with 50 ms CW laser (Figure 3f) and 5.24 ± 0.27 mV with 100 ms CW laser (Figure 3g). The depolarizations reached a maximum at 2.86 ± 8.55 ms before and 1.55 ± 4.78 ms after laser offset in the 50 ms and 100 ms conditions, respectively. At 3 ms after laser onset in all CW laser conditions, the averaged membrane response was −0.042 ± 0.50 mV, almost the same as the baseline, showing that the 3 ms of PT heating is not enough to induce obvious membrane depolarization.

Besides the difference in amplitudes and variations, distinct characteristics were shown in the rising and falling phases of the membrane responses to the PA and PT stimulations. Membrane depolarizations triggered by the pulsed ns laser showed a much faster responding and decay rate compared to those by the CW laser. To better compare the properties of the subthreshold events, we analyzed the rising rate at the beginning of the responses, and the time constant of the recovering periods by a single exponential fit in each laser condition (Figure 3h). In the pulsed laser condition, subthreshold events had an average rising rate of 0.97 ± 0.49 mV ms^−1^. In 10 ms, 50 ms, and 100 ms CW laser conditions, the rising rates were 0.23 ± 0.18 mV ms^−1^, 0.11 ± 0.049 mV ms^−1^, and 0.18 ± 0.086 mV ms^−1^, respectively. ANOVA test showed a significantly larger membrane depolarization speed in the PA condition compared to all of the PT conditions (^****^ *p* < 0.0001, Figure 3i).

At the decay phase, a time constant of 6.34 ± 5.09 ms suggested a much faster decay phase of PA‐triggered subthreshold depolarizations compared to the PT‐triggered ones (30.02 ± 8.49 ms for 10 ms, 34.98 ± 13.1 ms for 50 ms, 39.65 ± 9.56 ms for 100 ms). Notably, membrane responses in PA conditions demonstrated a two‐phase decay, instead of a single exponential component, which indicates the activation of active membrane components, namely the opening of ion channels,^[^ ^26^ ^]^ in addition to passive responses of the lipid membrane. Accordingly, the statistical summary suggests a significant difference in the decay time between the PA and PT‐ triggered subthreshold depolarizations (^****^ *p* < 0.0001, Figure 3j). No significant difference was found within PT conditions in the rising rate (n.s., *p* = 0.076, Figure 3i) or the time constant (n.s., *p* = 0.115, Figure 3j), indicating they all resulted from temperature‐induced capacitance responses on the cell membrane.

Collectively, these results show that PA and PT have triggered distinct electrical responses. In consistence with calcium imaging data, our patch data supports that thermal effect did not play a major role in the membrane responses to the pulsed laser and that PA should trigger the membrane depolarizations through a non‐thermal mechanism.

### 2.5 Blocking Mechanosensitive Ion Channels Effectively Suppresses PA Stimulation

The distinct membrane responses to PA versus PT stimulation inspired us to investigate the molecular mechanisms. Previous studies have suggested that mechanosensitive ion channels play a significant role in ultrasound neurostimulation.^[^ ^3^ ^]^ Thus, we hypothesized that PA alters the mechanical properties of the plasma membrane and subsequently induces the activation of mechanosensitive channels. These channels accumulate ionic currents to form membrane depolarization and subsequently activate voltage‐gated sodium channels to induce action potentials. To test this hypothesis, the contribution of mechanosensitive channels and voltage‐sensitive sodium channels in neuromodulation was assessed by pharmacological inhibition of channel activity.

To compare the PA stimulation results with blockers of different ion channels, a control experiment on GCaMP6f‐neurons without any pharmacological treatment was performed. Syn‐driven GCaMP6f, a Ca^2+^ sensor, was delivered to neurons via AAV9 viral vector at 4 days in vitro (See Methods). By applying a 3 ms pulse train of ns pulses with an energy of 24 µJ per pulse to an FE of 100 µm in diameter, the PA signal generated successfully stimulated the surrounding neurons with an averaged Max Δ*F*/ *F*_0_ =  122.1 ± 75.2%. This result is considered as the baseline for other neuron groups with different kinds of blockers under the same PA stimulation.

**Figure 4:**
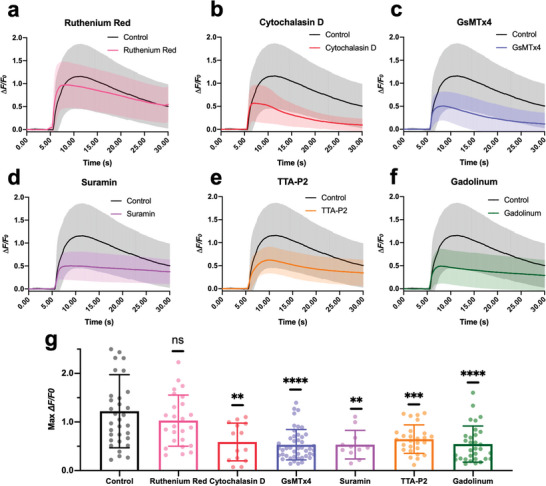
Blocking mechanosensitive ion channels affects PA stimulation of cortical neurons. a‐f. Calcium traces of neurons treated with blockers (Ruthenium Red: pink, *n* = 25. Cytochalasin D: red, *n* = 14. GsMTx4: blue, *n* = 45. Suramin: purple, *n* = 11. TTA‐P2: orange, *n* = 27. Gadolinum: green, *n* = 31. Control: Black, *n* = 33.) upon FE photoacoustic stimulation. Shaded areas: SD. Laser condition: 1030 nm, 24 µJ/pulse,  3 ms,  1.7 kHz. g) Statistics of the max *ΔF/F_0_* under varied treatments. *t‐test* n.s. not significant. ^****^ *p* < 0.0001, ^***^ *p* < 0.001, ^**^ *p* < 0.01.

First, to evaluate the involvement of thermosensitive ion channels, ruthenium red was applied to the neurons to block TRPV1, TRPV2, and TRPV4 channels.^[^ ^9^, ^27^ ^]^ As shown in **Figure** **4**a,g, no significant difference was observed compared to the control group (n.s., *p* = 0.28), indicating that these channels were not involved under this PA condition. Similar findings were also reported in transducer‐based ultrasound stimulation.^[^ ^3^ ^]^

Next, to alter the mechanical properties of the neurons, cytochalasin D was added to the neuron culture to inhibit the membrane ruffling by depolymerizing the actin cytoskeleton.^[^ ^28^ ^]^ The Max *ΔF/F_0_* decreased by 63.3% (^**^ *p* = 0.0047) (Figure 4b,g), indicating that the elastic modulus is important during the acoustic‐induced membrane distortion. Next, the peptide inhibitor GsMTx4, which blocks Piezo1 and TRPC1 channels, was used. The Max *ΔF/F_0_* of neurons showed a decrease of 69% in response to the FE‐based PA stimulation (^****^ *p* < 0.0001) (Figure 4c,g), indicating that the Piezo1 and/or TRPC1 channels play a key role during this process.

G protein‐coupled receptors (GPCRs) are sensory molecules reported to be important for mechano‐transduction in vasculature as shear stress sensors.^[^ ^29^ ^]^ To investigate whether the GPCRs were activated, suramin was used to block GPCR signaling (Figure 4d,g). The Max *ΔF/F_0_* decreased by 68.9% (^**^ *p* = 0.0053), suggesting the shear stress promoted the signaling of GPCRs for cell activation. It is also worth noting that, in the recent work using focused ultrasound for neurostimulation, GPCRs were shown to be not involved in the stimulation process.^[^ ^3^ ^]^ This discrepancy might originate from the difference in acoustic wave propagation. In the focused‐ultrasound study, the spherical focal area of the acoustic wave with a 5 mm diameter could cover the entire cell culture and be regarded as a planar wave. While in our FE work, the generated acoustic field has a sub‐millimeter diameter and propagates omnidirectionally, denoting a point source. Thus, shear stress was likely to be present in the lateral wave propagation of the FE‐generated ultrasound field.

Besides, *L*‐type, *n*‐type, *T*‐type, and *p*‐type calcium channels have been shown to be mechanically sensitive under various conditions.^[^ ^30^ ^]^ Recently, voltage‐gated *T*‐type calcium channels were reported to be downstream amplifiers for ultrasound neuromodulation.^[^ ^3^ ^]^ To validate this, we treated the cells with the selective blocker TTA‐P2, which suppressed the Max *ΔF/F_0_* by 57.4% (^***^ *p* = 0.0004) (Figure 4e,g). Thus, voltage‐gated *T*‐type calcium channels are likely activated during PA stimulation.

Lastly, we tested the effect of Gadolinium(III), which has been reported as a nonspecific agent that blocks mechano‐gated channels via changing the deformability of the lipid bilayer.^[^ ^31^ ^]^ As shown in Figure 4f, the FE‐induced calcium activities were significantly inhibited. Statistical results (Figure 4g) show that Gadolinium(III) resulted in a *ΔF/F_0_* decrease by 67.5% (^****^ *p* < 0.0001). These data collectively demonstrate the involvement of mechanosensitive ion channels in PA stimulation.

### 2.6 Overexpressing TRPC1/TRPP2/TRPM4 Boosts the Calcium Signal Upon PA Stimulation

**Figure 5:**
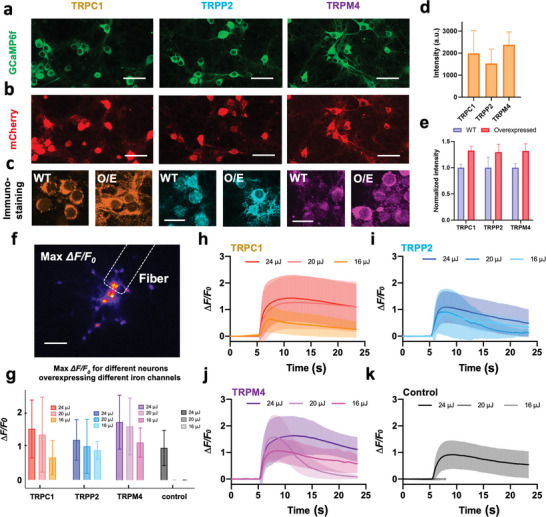
Response of neurons to PA stimulation after overexpression of mechanical sensitive ion channels. a) Neurons expressing GCaMP6f as a calcium indicator. b) Co‐expressing of mCherry with the TRPC1/TRPP2/TRPM4 ion channels. Scale bars: 50 µm. c) Immunostaining of ion channels in the wild type (WT) group and overexpression (O/E) groups. Scale bars: 20 µm. d) Statistics of the mCherry signal intensity. *n* = 3 for each group. e) Statistics of the immunostaining signal intensity. *n* = 3 for each group. f) Representative max *ΔF/F_0_* contrast imaging of tapered FE‐based PA stimulation. Dashed line: location of the tapered FE. Scale bar: 100 µm. g) Analysis of the calcium responses of neurons overexpressing ion channels upon FE stimulation with varied laser pulse energy. Error bars: SD. h–k) Calcium traces of neurons overexpressing TRPC1 (*n* = 87) / TRPP2 (*n* = 28) / TRPM4 channels (*n* = 26) and control group (*n* = 55, recording was ended early due to no response observed) upon FE stimulation with varied laser pulse energy. Shaded areas: SD. Control: wild type.

According to an earlier report that ultrasound excites neurons via the activation of endogenous mechanosensitive ion channels, including TRPP2 and TRPC1, as well as the calcium‐dependent amplifier TRPM4 channel,^[^ ^3^ ^]^ we overexpressed these three ion channels to identify their roles in PA stimulation. The gene constructs for TRPC1, TRPP2, and TRPM4 ion channels were overexpressed in GCaMP6f labeled neurons under a hSyn promoter (**Figure** **5**a). The fluorescent protein mCherry was co‐expressed as an expression indicator (Figure 5b). To further quantify the overexpression of ion channels, immunofluorescent labeling was performed in the wild type, as the control group and the overexpression groups. As shown in Figure 5c–e, the signals for TRPC1, TRPP2, and TRPM4 channels in the overexpression groups are 33 ± 8%, 30 ± 15%, and 32 ± 14% higher than the wild‐type groups, respectively, suggesting successful and comparable overexpression.

Next, the FE‐based PA stimulation was performed on neurons overexpressing specific ion channels. A 3 ns pulsed laser at 1030 nm and a 1.7 kHz repetition rate was used to deliver laser pulses of 3 ms duration to a FE with a 100 µm tip diameter (Figure 5f). Varied laser pulse energy of 16, 20, and 24 µJ were applied to test the neuron activation threshold (Figure 5h–k). As shown in Figure 5h,i, the PA stimulation of neurons overexpressing TRPC1 and TRPP2 evoked substantially larger calcium activities (TRPC1: Max *ΔF/F_0_* = 151.8 ± 84.9%, TRPP2: Max *ΔF/F_0_* = 119.4 ± 59.7%) compared to the control group (Max *ΔF/F_0_* = 90.3 ± 50.1%) with pulse energy of 24 µJ. Meanwhile, under the pulse energy of 16 or 20 µJ, the TRPC1 and TRPP2 groups could be stimulated (TRPC1: Max *ΔF/F_0_* at 16 µJ = 67.3 ± 49.9%, Max *ΔF/F_0_* at 20 µJ = 134.8 ± 110.4%; TRPP2: Max *ΔF/F_0_* at 16 µJ = 88.9 ± 26.2%, Max *ΔF/F_0_* at 20 µJ = 100.6 ± 79.5%), while no activity was observed in the control group, indicating that the overexpressing TRPC1 and TRPP2 channels increased the mechano‐sensitivity of the neurons (Figure 5g). Likewise, neurons overexpressing the TRPM4 channel showed increased calcium response and a lower stimulation threshold (Max *ΔF/F_0_* at 24 µJ = 172.5 ± 79.2%, Max *ΔF/F_0_* at 20 µJ = 159.9 ± 83.8%, Max *ΔF/F_0_* at 16 µJ = 112.5 ± 43.1%), validating the involvement of TRPM4. It is conceivable that TRPM4 serves as the downstream calcium‐dependent amplifier even though itself is not mechanosensitive (Figure 5j). Collectively, these results demonstrate the involvement of TRPC1, TRPP2, and TRPM4 channels in PA stimulation.

### 2.7 The Optocapacitive Mechanism is Not Involved in PA Stimulation

To test the possible involvement of the optocapacitive mechanism in PA stimulation, we harnessed an 80 MHz femtosecond (fs) laser to FE to deliver a rapid temperature increase in a 3 ms period.

The Newton's law of heating states that *mC_s_(ΔT) = Q_abs_*, where *m* is the mass and *C_s_* is the heat capacity. In the same period of 3 ms, if the total heat *Q_abs_* is the same, Δ*T*, the total temperature rise should be the same over the same period. For each pulse, the temperature rising rate is proportional to the heat transfer rate, which is linear to the pulse peak power. In our case, we used a nanosecond laser with a 4.23 kHz repetition rate, 3 ns pulse duration, and a fs laser with a 80 MHz repetition rate, and 140 fs pulse duration with the same averaged power of 120 mW. According to P_peak_ = P_ave_/(R_f_*τ), where *R_f_* and *τ* are the laser repetition frequency and pulse duration, respectively, the peak power of the fs laser condition is 1.13 times that of the ns laser. Based on this rationale, we expect that the fs laser delivers a similar temperature increase rate as the ns laser does and can be used to evaluate the optocapacitive contribution in the PA stimulation. Meanwhile, with the same peak power and averaged power, the fs laser has a large repetition rate of 80 MHz, corresponding to a small pulse energy (1.5 nJ pulse^−1^). Since the pressure from the photoacoustic effect is related to the pulse energy, the PA effect from this fs laser condition is negligible.

Experimentally, under the fs laser condition, with the same burst duration of 3 ms, the FE failed to stimulate neurons (Figure S6, Supporting Information black line, *n* = 24). In contrast, under the same burst duration, the ns laser can efficiently evoke neurons with a Max *ΔF/F_0_* larger than 5% (Figure S6, Supporting Information red line, *n* = 10). This result indicates that the rapid temperature increase that occurred during the fs laser condition is insufficient to stimulate neurons. Under the ns laser condition, despite the presence of the same temperature increase rate, it is the PA effect that successfully stimulated the neurons.

## 3 Discussion

In this work, using a unified fiber emitter of heat and ultrasound, we compared the laser energy needed for PA and PT stimulation. The laser energy needed for PA stimulation was shown to be 1/40 of that for PT stimulation. This substantial difference demonstrates that the PT effect associated with the PA process alone is not sufficient to trigger neurons; instead, the generated ultrasound is the key factor for neurostimulation. Practically, the lower energy required for PA stimulation is advantageous in two perspectives. First, it reduces the potential risk of thermal damage in neural tissues. Second, the lower energy requirement opens up the potential of applying PA stimulation in deeper tissue as it can afford more energy loss due to scattering.

The whole‐cell patch recording has long served as the golden standard for studying neuronal responses to stimuli. However, measuring cellular responses under transducer‐produced ultrasound has proven exceptionally challenging due to the widespread interference of ultrasound with the patch, and only a few studies have reported measurements under pressures up to 200 kPa.^[^ ^32^ ^]^ Our study demonstrates that whole‐cell patch measurements of individual cultured neurons are possible under high‐precision PA and PT stimulation. Our findings showed that PA‐induced membrane depolarizations had significantly faster rising and falling rates, indicating the involvement of ion channel activation. In contrast, the depolarization amplitudes in PT stimulation were sensitive to temperature change, with the rising and falling rate of depolarizations aligned with the rate of temperature change. These results suggest a predominant role of a heat‐induced capacitance mechanism in PT stimulation,^[^ ^10^, ^11^ ^]^ rather than the activation of temperature‐sensitive ion channels. Collectively, these findings highlight distinct molecular mechanisms underlying the depolarization responses to PA and PT stimulations.

We further investigated the molecular mechanisms underlying PA modulation. Since the PA devices generate acoustic waves in the ultrasonic range, it is conceivable that PA neurostimulation shares similar mechanisms with ultrasound neurostimulation. To date, several mechanisms for ultrasound stimulation have been proposed, including local temperature increase,^[^ ^33^ ^]^ transient sonoporation,^[^ ^34^ ^]^ intramembrane cavitation,^[^ ^35^ ^]^ and activation of mechanosensitive ion channels.^[^ ^3^, ^36^ ^]^ Among these possible mechanisms, activation of mechanosensitive ion channels has been the most extensively studied hypothesis for acoustic neuromodulation. In an oocyte membrane system, Kubanek et al. recorded transmembrane currents from individually expressed mechanosensitive ion channels including TREK‐1, TREK‐2, TRAAK, and Na_v_1.5.^[^ ^36a^ ^]^ A later study by Kubanek et al. further identified MEC‐4, an ion channel for a touch sensation, was crucial for ultrasound‐modulated responses in Caenorhabditis elegans.^[^ ^36b^ ^]^ In addition, the overexpression of TRP‐4, a TRPN family channel has been shown to enhance ultrasound modulation in Caenorhabditis elegans as well.^[^ ^37^ ^]^ Employing calcium imaging, Gaub et al. investigated the neuronal response to pure mechanical stimuli using atomic force microscope cantilever.^[^ ^38^ ^]^ They identified the force and pressure required for both transient and sustained activation. The contribution of various mechanosensitive ion channels has also been explored through pharmacological manipulation. Using calcium imaging, Yoo et al. examined the activation of various mechanosensitive ion channels upon ultrasound stimulation and identified the key contribution of three ion channels: TRPP2, TRPC1, and Piezo1.^[^ ^3^ ^]^ The proposed downstream molecular pathway involves calcium amplification by TRPM4 and voltage‐gated calcium channels. In this work, we showed PA stimulation of primary cortical neurons through specific calcium‐selective mechanosensitive ion channels with the assistance of calcium amplifier channels and voltage‐gated channels. As our key findings, pharmacological inhibition of specific ion channels leads to reduced responses, while over‐expressing TRPC1, TRPP2, and TRPM4 channels results in stronger stimulation. Collectively, these results shed new insights into the mechanism of PA and ultrasound neurostimulation.

Further investigations of cellular mechanisms of PA stimulation are needed. While our findings indicate the energy threshold for PA stimulation is much less than PT stimulation, the underlying mechanism remains to be elucidated. The excitability of the mechanosensitive ion channels and thermosensitive ion channels needs to be studied and compared. The involvement of other mechanisms, such as sonoporation, needs to be investigated. Further investigations are warranted to elucidate the underlying cellular and molecular mechanisms driving the remarkable efficacy of PA as a neuromodulation method.

## 4 Experimental Section

### FE Fabrication and Characterization

For Ca^2+^ imaging of PA and PT neuromodulation, a multimode fiber (FT200EMT, Thorlabs, Inc., NJ, USA) with 200 µm in diameter was used. The PA coating was composed of candle soot and PDMS. Candle soot was chosen as the absorber due to its great absorption coefficient. The multimode optical fiber was exposed to the candle flame for around 3–5 s until the fiber tip was fully coated, with the thickness of the candle soot controlled by the deposition time.^[^ ^21^ ^]^ To prepare PDMS, the silicone elastomer (Sylgard 184, Dow Corning Corporation, USA), was carefully dispensed into a container to minimize air entrapment, and then mixed with the curing agent in a ratio of 10:1 by weight. A nanoinjector deposited the prepared PDMS onto the tip of the candle‐soot‐coated fiber and thus formed a layered structure.^[^ ^39^ ^]^ The position of the fiber and the nanoinjector were both controlled by 3D manipulators for precise alignment, and the PDMS coating process was monitored in real time under a lab‐built microscope. The coated fiber was then cured overnight at room temperature.

For stimulation performed with whole cell patch with single‐cell stimulation, tapered FE was prepared using multimode optical fibers as previously described.^[^ ^26^ ^]^ For pharmacological blocking and ion channel studies, the same type of tapered FE was used.

To characterize the photoacoustic signal generated by the FE, a customized and compact passively Q‐switched diode‐pumped solid‐state laser (1030 nm, 3 ns, 100 µJ, repetition rate up to 10 kHz, RPMC, Fallon, MO, USA) served as the excitation source. The laser was first connected to an optical fiber through a customized fiber jumper (SMA‐to‐SC/PC, 81% coupling efficiency) and then connected to the FE with a SubMiniature version A (SMA) connector. To adjust the laser power, fiber optic attenuator sets (multimode, varied gap of 2/4/8/14/26/50 mm, SMA Connector, Thorlabs, Inc., NJ, USA) were used. A needle hydrophone (ID. 40 µm; OD, 300 µm) with a frequency range of 1–30 MHz (NH0040, Precision Acoustics Inc., Dorchester, UK) was utilized for the acoustic measurement. Both the fiber and the hydrophone were aligned under the microscope (Figure S1, Supporting Information). The acquired signal was processed with an ultrasonic pre‐amplifier (0.2–40 MHz, 40 dB gain, Model 5678, Olympus, USA) and a digital oscilloscope (DSO6014A, Agilent Technologies, USA). The distance between the FE tip and hydrophone was controlled using a 4‐axis micro‐manipulator (MC1000e controller with MX7600R motorized manipulator, Siskiyou Corporation, USA) with a controllable motion of 0.2 µm. The distance was measured using a widefield microscope with a 20× objective. Both the FE tip and hydrophone tip were immersed in degassed water dropped on a cover glass. The pressure values were calculated based on the calibration curve obtained from the hydrophone manufacturer. The frequency data was obtained through the Fast Fourier Transform (FFT) using MATLAB 2020a.

### Neuron Culture with GCaMP6f / mCherry Expression

All experimental procedures complied with all relevant guidelines and ethical regulations for animal testing and research established and approved by Institutional Animal Care and Use Committee (IACUC) of Boston University (PROTO201800534). Primary cortical neurons were isolated from embryonic day 15 (E15) Sprague−Dawley rat embryos of either sex (Charles River Laboratories, MA, USA). Dissociated cells were washed and triturated with 10% heat‐inactivated fetal bovine serum (FBS, Atlanta Biologicals, GA), 5% heat‐inactivated horse serum (HS, Atlanta Biologicals, GA), 2 mM Glutamine‐Dulbecco's Modified Eagle Medium (DMEM, Thermo Fisher Scientific Inc., MA), and cultured in cell culture dishes (100 mm diameter) for 30 min at 37 °C to eliminate glial cells and fibroblasts. The supernatant containing neurons was collected and seeded on poly‐D‐lysine coated cover glass and incubated in a humidified atmosphere containing 5% CO_2_ at 37 °C with 10% FBS + 5% HS + 2 mM glutamine DMEM. After 16 h, the medium was replaced with Neurobasal medium (Thermo Fisher Scientific Inc., MA) supplemented with 2% B27 (Thermo Fisher Scientific Inc., MA), 1% N2 (Thermo Fisher Scientific Inc., MA), and 2 mM glutamine (Thermo Fisher Scientific Inc., MA). Half of the medium was changed with the fresh medium every 3 days, and neurons were used for stimulation experiments after 10–14 days after seeding.

For calcium imaging, Syn‐driven GCaMP6f as a calcium sensor was delivered to neurons via AAV9 viral vector transfection (Addgene, pAAV.Syn.GCaMP6f.WPRE.SV40, 1E10 vp/dish) at day 4 after seeding. To measure temperature change during photoacoustic and photothermal stimulation, mCherry as a temperature sensor was delivered to neurons via AAV9 viral vector transfection (Addgene, pAAV‐hSyn‐mCherry, 1E10 vp/dish). The viral particles were added to neurons at day 3 after seeding. The whole media was replaced with the fresh media at day 4 after seeding, and the cells were maintained for 6–10 additional days.

### Calcium Imaging of FE‐Induced Neuron Stimulation

Calcium fluorescence imaging was performed on a lab‐built wide‐field fluorescence microscope. The microscope was based on an Olympus IX71 microscope frame, with a 20X air objective (UPLSAPO20X, 0.75NA, Olympus), illuminated by a 470 nm LED (M470L2, Thorlabs) and a dichroic mirror (DMLP505R, Thorlabs). A 3‐D micromanipulator (Thorlabs, Inc., NJ, USA) positioned the FE at an angle of 45° to the cells, maintaining a distance of ∼50 µm between the FE tip and the culture. Image sequences were acquired with a scientific CMOS camera (Zyla 5.5, Andor) at 20 frames per second. The fluorescence intensity analysis was performed using ImageJ (Fiji).

In vitro PA neurostimulation experiments were performed using a Q‐switched 1 030 nm nanosecond laser (Bright Solution, Inc. Calgary Alberta, CA). In vitro PT neurostimulation experiments were performed using a CW diode pumped laser (Cobolt Rumba 05‐01 series, Sweden).

### Temperature Measurement In Vitro Using mCherry Fluorescence

Fluorescence imaging of mCherry was conducted on a lab‐built wide‐field fluorescence microscope (the same system used for calcium imaging). With a 20X air objective (UPLSAPO20X, 0.75NA, Olympus), neurons were illuminated by a white LED with a mCherry filter cube (562/40 excitation, 641/75 emission, and a dichroic mirror, MDF‐MCHC, Thorlabs). FE was precisely positioned using a 3‐D micromanipulator (Thorlabs, Inc., NJ, USA). Image sequences were acquired with a scientific CMOS camera (Zyla 5.5, Andor) at 20 frames per second. The fluorescence intensity analysis was performed using ImageJ (Fiji).

### Gene Overexpression of Ion Channels in Cultured Neurons

As described in the previous work,^[^ ^3^ ^]^ the mouse TRPP2 (GenBank: BC053058), TRPM4 (GenBank: BC096475), and human TRPC1 (GenBank: Z73903.1) genes were synthesized commercially (Integrated DNA Technologies) and cloned upstream of an internal ribosome entry site (IRES2) and mScarlet (TRPC1, TRPP2) or mRuby3 (TRPM4) gene. The construct was inserted into the lenti‐backbone. The viral particles were added to neurons at 3 days in vitro (1E9 vp/sample) and maintained for 10 days. hSyn‐driven mCherry was inserted into the lenti‐backbone by Gibson assembly to confirm the gene expression. The viral particles were added to neurons at 3 days in vitro (1E9 vp/sample), whole media was replaced with the fresh supplemented Neurobasal media at 4 days in vitro, and the cells were maintained for 10 additional days.

### Immunostaining Characterization of Ion Channel Expression Levels

For immunostaining, primary neurons were fixed using ice‐cold paraformaldehyde (4% in PBS, VWR) for 10 min at 4 °C, and washed with PBS twice. Nonspecific biding was blocked by 6% bovine serum albumin (Sigma) for 30 min at room temperature, and cells were washed in PBS. Primary antibody anti‐TRPC1 (1:200, Alomone Labs), anti‐TRPM4 (1:200, Alomone Labs) and anti‐TRPP2 (1:200, Alomone Labs) were diluted in 1.5% bovine serum albumin, and incubated with cells at 4 °C overnight. After washing with PBS for 3 times, secondary antibodies (Alexa Fluor 488 (1:500, Invitrogen) diluted in 1.5% BSA were added to neurons for 1 h at 37 °C. Cells were washed with PBS, and imaged using a confocal microscope (FV3000, Olympus).

### Pharmacological Treatments with Chemical Blockers and Peptide Inhibitors of Ion Channels

The following blockers were added to medium and incubated with cells for 4 h before stimulating cells with optoacoustic: Ruthenium red (final conc.: 1 µM) was used before PA stimulation to block TRP channels (TRPV1, 2, 4). Actin filaments were depolymerized by their specific inhibitors, cytochalasin D (final conc.: 10 µM). GsMTx4 was added to medium (final conc.: 10 µM) to inhibit Piezo1 and TRPC1 channels. To inhibit GPCRs, suramin was added (final conc.: 60 µM). TTA‐P2 (final conc.: 3 µM) was added to block T‐type calcium channels. Gadolinium was applied to nonspecifically block the mechanosensitive ion channels (final conc.: 20 µM).

### Electrophysiology and Action Potential Analysis

Membrane potentials were recorded in current clamp mode with an Axopatch 200B amplifier (Axon Instruments, Union City, CA, USA) and digitized with an NI 6251 board (National Instruments, Austin, TX, USA). Signals were low‐pass filtered at 10 kHz and sampled at 5 kHz. Cells were recorded at a holding potential of −70 mV in a bath solution (140 mM NaCl, 3 mM KCl, 1.5 mM MgCl_2_, 2.5 mM CaCl_2_, 11 mM glucose and 10 mM HEPES, pH 7.4). Data analysis only included cells with resting membrane potentials between −60 to ‐70 mV. The bath solution, heated to 30 °C, was circulated in the petri dish during the whole recording period. The recording electrodes were filled with a K^+^‐based internal solution (135 mM K^+^‐gluconate, 5 mM NaCl, 2 mm MgCl_2_, 10 mM HEPES, 0.6 mM EGTA, 4 mM Mg^2+^‐GTP, 0.4 mM Ma^+^‐ATP) and had a resistance ranging from 5 to 10 MΩ. Data were analyzed and visualized with IGOR PRO (Wavemetrics, Lake Oswego, OR, USA). The threshold of an AP was defined as the membrane potential when the dv/dt first exceeds 10 V s^−1^.

## Conflict of Interest

JXC and CY claim COI with Axorus which did not support this work. Other authors claim no COI.

## Author Contributions

GC, LS, FY, J‐XC, and CY performed drafting and revising the manuscript. GC conducted the PA and PT comparison under calcium imaging. LS conducted the pharmacological and genetic studies. FY conducted the experiment of patch clamp recording. This project was performed under the critical guidance of J‐XC and CY. ZD, CM, and DJ helped with the experiments. All authors have read and approved the manuscript.

## Supporting information

Supporting Information

Supplemental Movie 1

## Data Availability

The data that support the findings of this study are available from the corresponding author upon reasonable request.
