# Supplementary material for: High‐Precision Photoacoustic Neural Modulation Uses a Non‐Thermal Mechanism
Source: Adv Sci (Weinh). 2024 Jun 26;11(32):2403205. doi: 10.1002/advs.202403205 (PMC11348214; doi:10.1002/advs.202403205)
Supplement: Supplementary file 1 — Supporting Information [file ADVS-11-2403205-s001.docx]

**Supplementary Figures**

**High-precision optoacoustic neural modulation uses a non-thermal mechanism**

Guo Chen^1†^, Feiyuan Yu^1†^, Linli Shi^2^, Carolyn Marar^3^, Zhiyi Du^2^, Danchen Jia^1^, Ji-Xin Cheng^1, 3,4*^, Chen Yang^1, 2,4*^

^1^ Department of Electrical and Computer Engineering, Boston University, Boston, MA, 02215, United States

^2^ Department of Chemistry, Boston University, Boston, MA, 02215, United States

^3^ Department of Biomedical Engineering, Boston University, Boston, MA, 02215, United States

^4^ Photonics Center, Boston University, Boston, MA, 02215, United States

* Corresponding authors jxcheng@bu.edu; cheyang@bu.edu

^†^ These authors contributed equally to this work

**
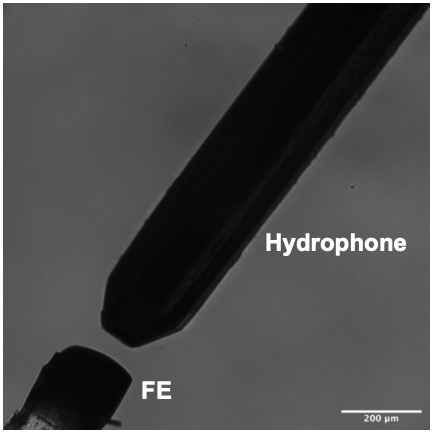
**

**Figure S1. Measurement of fiber-emitter generated photoacoustic signal by a needle hydrophone.** Left: FE. Right: hydrophone.

**
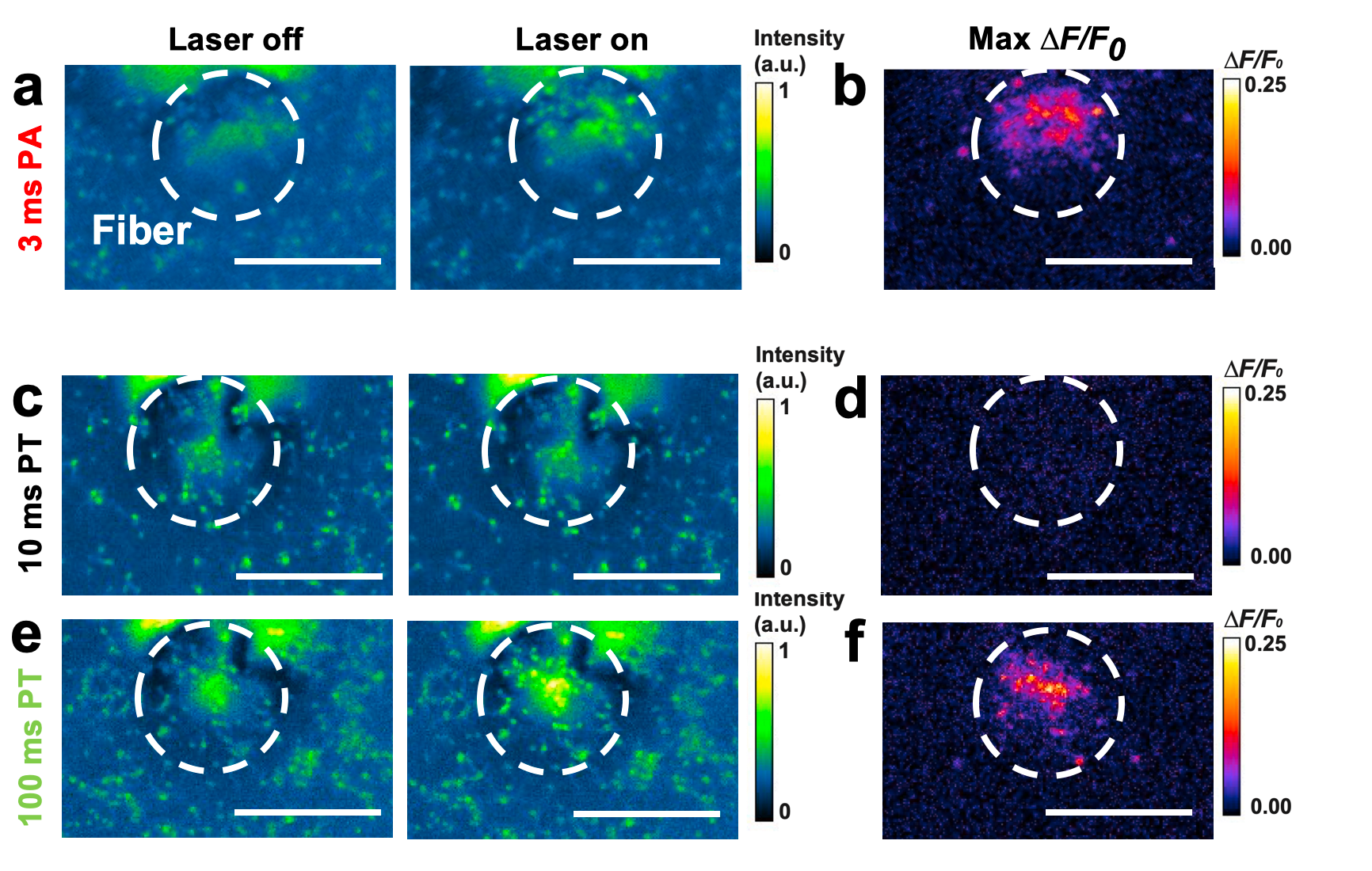
**

**Figure S2**. **Calcium imaging of PA and PT stimulated neurons.**  a-b. representative fluorescence imaging of OGD labeled neuron before / after laser is on. Laser condition: 3 ns pulsed laser, 120 mW, 3 ms burst duration. c-d. representative fluorescence imaging of OGD labeled neuron before / after laser is on. Laser condition: CW laser, 120 mW, 10 ms burst duration. e-f. representative fluorescence imaging of OGD labeled neuron before / after laser is on. Laser condition: CW laser, 120 mW, 100 ms burst duration.


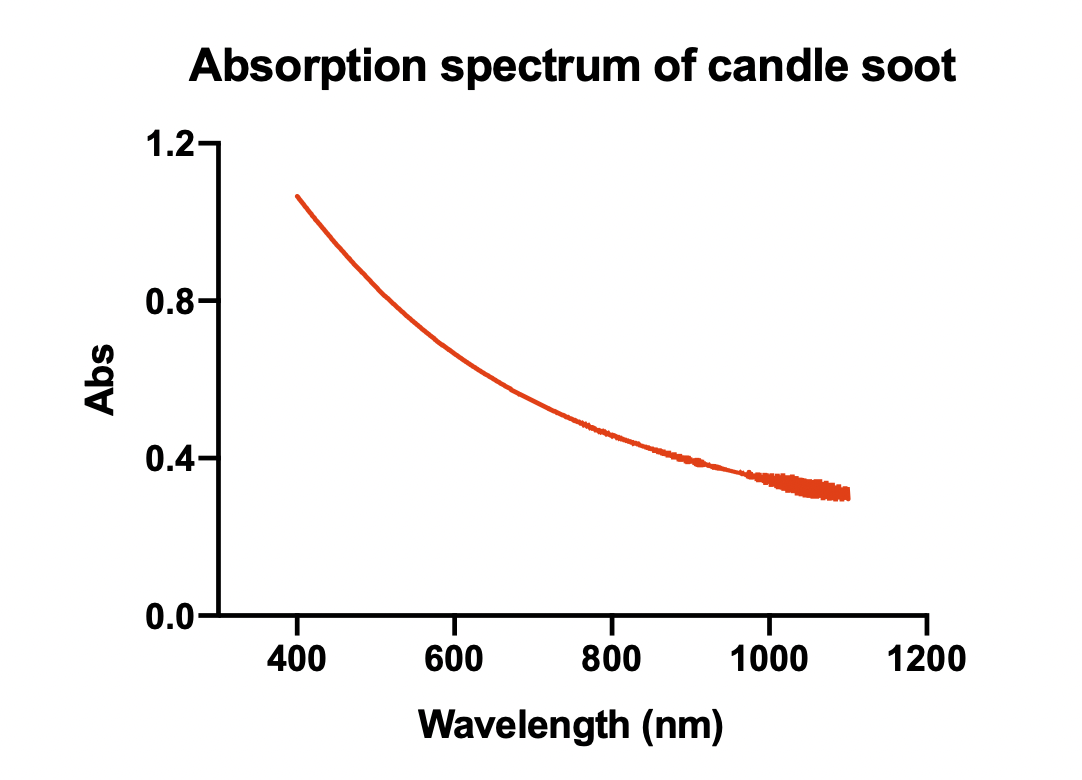


**Figure S3.** **UV-vis extinction spectrum of candle soot**. The absorption at 1064 nm and 1030 nm are similar.

**
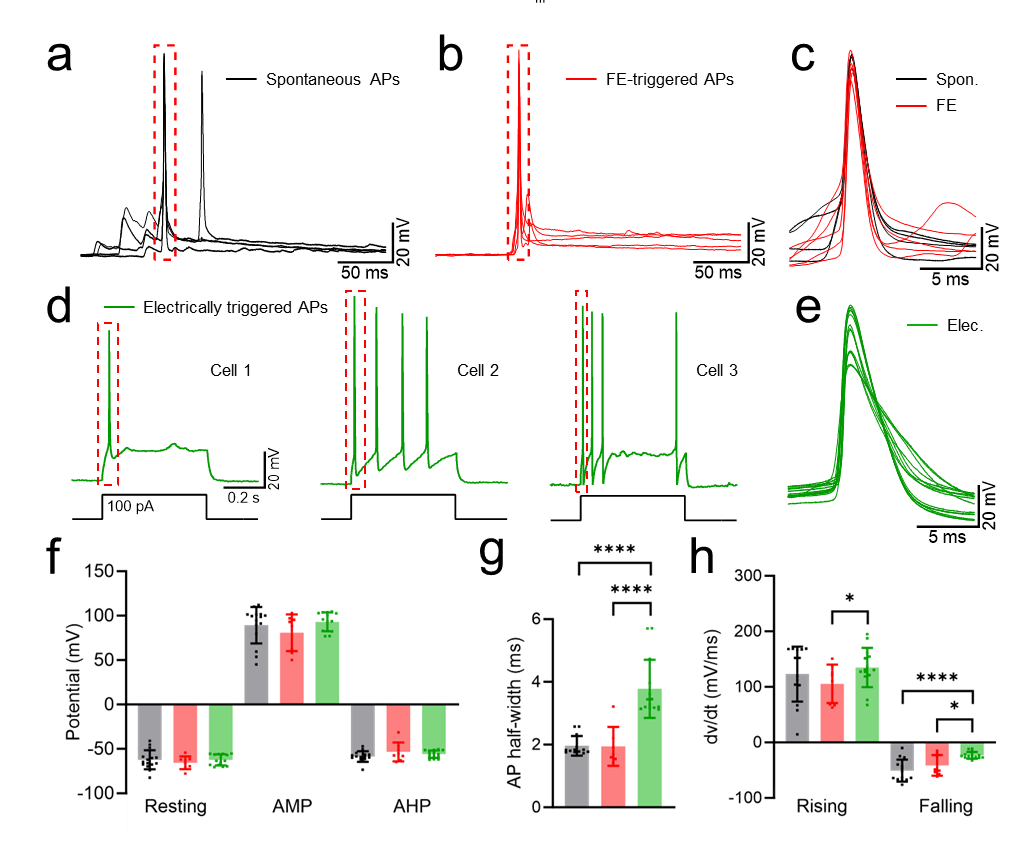
**

**Figure S4. PA-triggered APs showed similar characteristics as spontaneous APs.** a-b. Overlaying individual representative spontaneous (spon.) APs (a) and PA-triggered APs (b) recorded from a neuron in different trials. Red dashed boxes labelled the area further plotted in c. c. Peaks of the same APs in a and b in an expanded timescale. All traces were normalized to the same baseline and aligned by peaks. d. Variations in AP waveform responding to the same current injection in 3 different cells. e. Overlaying electrically stimulated APs. All traces were normalized to the same baseline and aligned by peaks. f-h. Summary of resting membrane potential (Spon. -62.30 ± 10.82 mV, FE -65.63 ± 7.157 mV, Elec -62.47 ± 6.25 mV), peak amplitude (AMP, Spon. 89.21 ± 20.55 mV, FE 80.78 ± 20.65 mV, Elec 93.04 ± 10.58 mV), afterhyperpolarization (AHP, spon. -58.65 ± 5.96 mV, FE -53.46 ± 10.62 mV, Elec -55.90 ± 4.23 mV), AP width at half-maximum peak amplitude (spon. 1.96 ± 0.31 ms, FE 1.94 ± 0.62 ms, Elec 3.78 ± 0.92 ms), maximum and minimum rate during rising (spon. 131.38 ± 39.96 mV/ms, FE 105.4 mV/ms, Elec 105.42 ± 34.73 mV/ms) and falling phase (spon. -53.91 ± 16.48 mV/ms, FE -41.37 ± 18.52 mV/ms, Elec -23.38 ± 6.27 mV/ms). Mean ± standard deviation was plotted. N = 15 from 3 cells for spontaneous and N = 8 from the same 3 cells for FE-stimulated trials. N=14 from 5 cells (including 2 cells in the spon. and FE groups) for electrical stimulation trials. Student *T-test*s. *p<0.05. ****p<0.0001.

**Laser pulses vs trigger signals**


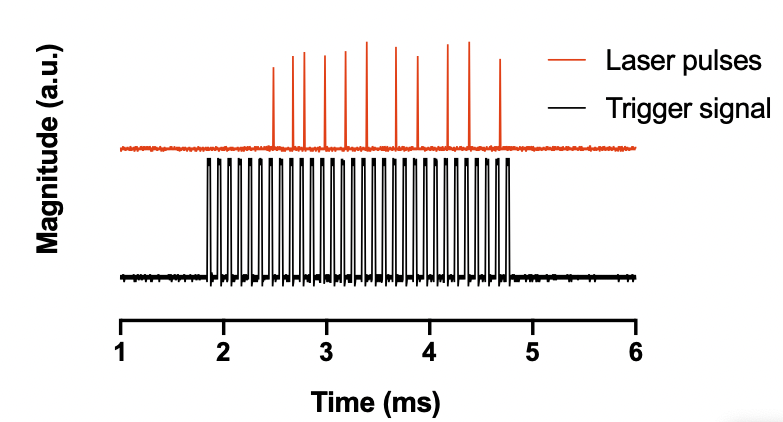


**Figure S5**. Laser pulses versus the trigger signal within 3 ms burst duration. There is a delay of around 0.6 ms after the laser receives the trigger signal.


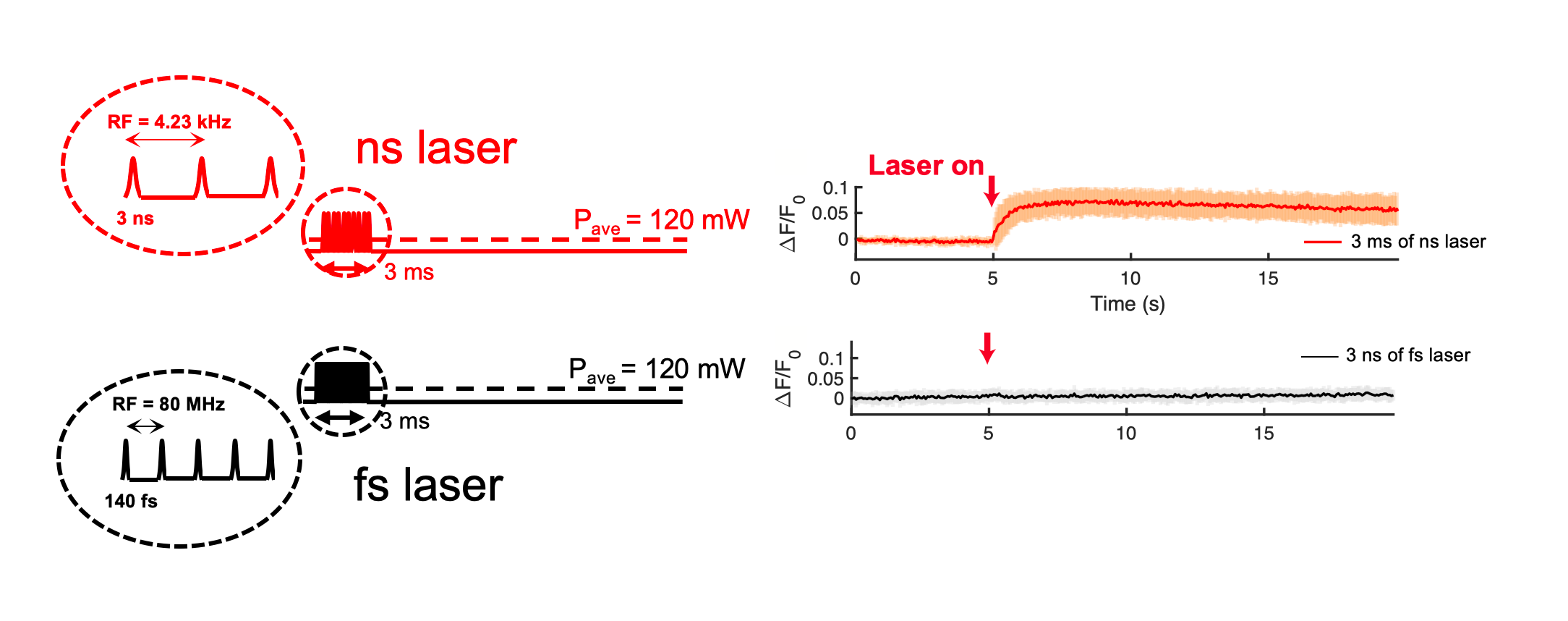


**Figure S6.** Stimulation of primary cortical neurons using a nanosecond pulsed laser (PA condition) and a femtosecond pulsed laser (optocapacitive condition), respectively. OGD488 fluorescence was recorded. N = 10 for PA condition and N =24 For optocapacitive condition.
